# 4-Ethylguaiacol modulates neuroinflammation and Th1/Th17 differentiation to ameliorate disease severity in experimental autoimmune encephalomyelitis

**DOI:** 10.1186/s12974-021-02143-w

**Published:** 2021-05-11

**Authors:** Wen-Tsan Weng, Ping-Chang Kuo, Dennis A. Brown, Barbara A. Scofield, Destin Furnas, Hallel C. Paraiso, Pei-Yu Wang, I-Chen Yu, Jui-Hung Yen

**Affiliations:** 1 Department of Microbiology and Immunology, Indiana University School of Medicine, 2101 E. Coliseum Boulevard, Fort Wayne, IN 46805 USA; 2 Department of Pharmaceutical Sciences, Manchester University College of Pharmacy, Natural and Health Sciences, Fort Wayne, IN USA; 3 Department of Anatomy, Cell Biology and Physiology, Indiana University School of Medicine, Fort Wayne, IN USA; 4 Graduate Institute of Brain and Mind Science, College of Medicine, National Taiwan University, Taipei, Taiwan

**Keywords:** MS/EAE, 4-EG, Microglia, Th1/Th17, Neuroinflammation, Blood-brain barrier

## Abstract

**Background:**

Multiple sclerosis (MS) is a progressive autoimmune disease characterized by the accumulation of pathogenic inflammatory immune cells in the central nervous system (CNS) that subsequently causes focal inflammation, demyelination, axonal injury, and neuronal damage. Experimental autoimmune encephalomyelitis (EAE) is a well-established murine model that mimics the key features of MS. Presently, the dietary consumption of foods rich in phenols has been reported to offer numerous health benefits, including anti-inflammatory activity. One such compound, 4-ethylguaiacol (4-EG), found in various foods, is known to attenuate inflammatory immune responses. However, whether 4-EG exerts anti-inflammatory effects on modulating the CNS inflammatory immune responses remains unknown. Thus, in this study, we assessed the therapeutic effect of 4-EG in EAE using both chronic and relapsing-remitting animal models and investigated the immunomodulatory effects of 4-EG on neuroinflammation and Th1/Th17 differentiation in EAE.

**Methods:**

Chronic C57BL/6 EAE and relapsing-remitting SJL/J EAE were induced followed by 4-EG treatment. The effects of 4-EG on disease progression, peripheral Th1/Th17 differentiation, CNS Th1/Th17 infiltration, microglia (MG) activation, and blood-brain barrier (BBB) disruption in EAE were evaluated. In addition, the expression of MMP9, MMP3, HO-1, and Nrf2 was assessed in the CNS of C57BL/6 EAE mice.

**Results:**

Our results showed that 4-EG not only ameliorated disease severity in C57BL/6 chronic EAE but also mitigated disease progression in SJL/J relapsing-remitting EAE. Further investigations of the cellular and molecular mechanisms revealed that 4-EG suppressed MG activation, mitigated BBB disruption, repressed MMP3/MMP9 production, and inhibited Th1 and Th17 infiltration in the CNS of EAE. Furthermore, 4-EG suppressed Th1 and Th17 differentiation in the periphery of EAE and in vitro Th1 and Th17 cultures. Finally, we found 4-EG induced HO-1 expression in the CNS of EAE in vivo as well as in MG, BV2 cells, and macrophages in vitro.

**Conclusions:**

Our work demonstrates that 4-EG confers protection against autoimmune disease EAE through modulating neuroinflammation and inhibiting Th1 and Th17 differentiation, suggesting 4-EG, a natural compound, could be potentially developed as a therapeutic agent for the treatment of MS/EAE.

**Supplementary Information:**

The online version contains supplementary material available at 10.1186/s12974-021-02143-w.

## Introduction

Multiple sclerosis (MS) is a progressive autoimmune disease characterized by the accumulation of pathogenic inflammatory immune cells in the central nervous system (CNS) that subsequently causes focal inflammation, demyelination, axonal injury, and neuronal damage [1, 2]. Experimental autoimmune encephalomyelitis (EAE) is a well-established murine model mimicking the key features of MS, making it a useful tool for MS research and drug discovery [1]. T cells specific for CNS antigens are reported to play a major role in the disease development of both MS and EAE [1, 2]. During the disease progression, autoreactive T cells augment CNS inflammation by secreting a variety of pro-inflammatory cytokines, including IFNγ, IL-17, and GM-CSF [3, 4]. In addition, the CNS-resident microglia (MG) have been shown to promote differentiation and reactivation of pathogenic T cells [5].

The dietary consumption of foods rich in phenols has been attributed to numerous health benefits, including lowering rates of chronic diseases, such as Alzheimer’s disease, Parkinson’s disease, diabetes, cardiovascular disease, and inflammation [6, 7]. One such molecule, 4-ethylguaiacol (4-ethyl-2-methoxyphenol, hereinafter referred to as 4-EG), is a phenolic compound with the molecular formula C_9_H_12_O_2_ and belongs to the class of organic compounds known as methoxyphenols [8, 9]. 4-EG and other methoxyphenols have been detected in wine and beer produced by the spoilage yeast *Brettanomyces* [10]. In addition, 4-EG can be detected in several different foods, such as green, orange, and yellow bell peppers; corn; sesame seeds; and coffee [9]. There has been growing evidence showing that phenolic compounds exert anti-inflammatory effects through inhibiting the production of TNFα, IL-6, IL-1β, and cyclooxygenase-2 [8, 11]. The anti-inflammatory effects of 4-EG were shown to be mediated through the inhibition of NFκB and inflammasome activation that led to the suppression of inflammatory cytokines in THP-1 human monocytic cells [8]. Furthermore, 4-EG was shown to induce nuclear factor erythroid 2-related factor 2 (Nrf2) signaling in LPS-treated THP-1 cells, and silencing Nrf2 significantly promoted NF-κB expression in the nucleus of LPS-stimulated THP-1 cells [8], suggesting that 4-EG-induced anti-inflammatory effects may be mediated through the induction of the Nrf2/heme oxygenase-1 (HO-1) pathway.

The induction of transcription factor Nrf2 is an antioxidant defense mechanism elicited to counteract oxidative stress in cells [12]. In addition, the induction of Nrf2 has been shown to provide potent anti-inflammatory effects [13–16]. The activity of Nrf2 is regulated via interaction with its repressor, Keap1. Under the basal conditions, Keap1 binds Nrf2, giving rise to a Keap1-associated ubiquitin ligase complex that results in ubiquitination and subsequent Nrf2 degradation. In response to oxidative stress, Nrf2 escapes Keap1-mediated repression and is translocated into the nucleus. In the nucleus, Nrf2 binds to the antioxidant response element, inducing the transcription of phase II detoxication enzymes, including NAD(P)H quinone dehydrogenase 1 (NQO1), glutamate-cysteine ligase catalytic subunit (GCLC), and the anti-inflammatory enzyme HO-1 [17, 18].

Currently, whether 4-EG exerts anti-inflammatory effects on modulating the CNS immune responses remains unknown. Thus, in this study, we explored the therapeutic effect of 4-EG on the CNS autoimmune disease, MS, using the animal model of EAE, and investigated the cellular and molecular mechanisms underlying the protective effects of 4-EG in EAE. Our results showed that 4-EG not only ameliorated the disease severity in C57BL/6 chronic EAE but also mitigated disease progression in SJL/J relapsing-remitting EAE. Further investigations revealed that 4-EG suppressed MG activation, inhibited Th1 and Th17 infiltration, and mitigated blood-brain barrier (BBB) disruption in the CNS of EAE. In addition, 4-EG suppressed Th1 and Th17 differentiation in the periphery of EAE. Finally, we identified that 4-EG promoted HO-1 expression in the CNS of EAE, and 4-EG-mediated HO-1 upregulation was further confirmed in primary MG, BV2 cells, and primary macrophages. Our findings provide the first evidence that 4-EG could be developed as a therapeutic agent for the treatment of MS/EAE.

## Material and methods

### Animals

Female C57BL/6 and SJL/J mice were purchased from the Jackson Laboratory (Bar Harbor, ME). Mice were housed and bred in the animal facility with controlled temperature, humidity, and a 12-h light/dark alternate cycle. All animal studies performed in this study were approved by the Purdue University Animal Care and Use Committee (PACUC) and conducted in strict compliance with the National Institutes of Health Guidelines for the Care and Use of Laboratory Animals.

### EAE induction

Female C57BL/6 and SJL/J mice (7–9 weeks) were immunized with MOG_35–55_ and PLP_139–151_, respectively, to induce EAE as previously described [15]. Briefly, for the chronic EAE model, C57BL/6 mice were subcutaneously immunized with MOG_35–55_ (200 μg per mouse) emulsified with complete Freund’s adjuvant (CFA) (MilliporeSigma, St. Louis, MO, USA) containing *Mycobacterium tuberculosis* H37 RA (BD, Sparks, MD, USA) with the final concentration of 2 mg/ml on day 0. For the relapsing-remitting EAE model, SJL/J mice were subcutaneously immunized with PLP_139–151_ (100 μg per mouse) emulsified with CFA containing *Mycobacterium tuberculosis* H37 RA with the final concentration of 2 mg/ml on day 0. Immunized C57BL/6 and SJL/J mice were then i.p. administered with 200 ng pertussis toxin (List Biological Labs, Campbell, CA, USA) on day 0 and day 2. For the treatment, 4-EG (MilliporeSigma) dissolved in phosphate-buffered saline (PBS) was i.p administrated to C57BL/6 EAE mice daily starting from day 3 post-immunization till day 30 post-immunization or to SJL/J EAE mice daily starting from the first remission phase (disease score ≤1.5) for 20 days. The vehicle was administered with an equal volume of PBS. EAE mice were monitored daily to assess disease scores. The disease scores were determined based on the following criteria: 0, no clinical signs; 1, limp tail or hind limb weakness; 2, limp tail and hind limb weakness; 3, partial hind limb paralysis; 4, complete hind limb paralysis; and 5, moribund state. EAE incidence, mortality, onset of disease, maximum score, and cumulative score (sum of disease scores) were also assessed. The investigators evaluating EAE disease scores were blinded to the EAE groups. A summarized table that describes the tissue distribution for different experiments is included in Table S1.

### CNS mononuclear cell isolation

C57BL/6 EAE mice were anesthetized and perfused with ice-cold PBS. The brains and spinal cords harvested from vehicle- and 4-EG-treated EAE mice were excised and homogenized with 1× HBSS buffer containing collagenase. Dissociated cells were then filtered through 70-μm nylon meshes and pelleted by centrifugation. After resuspension, cells were subjected to 30/70 Percoll density gradient, and the mononuclear cells were harvested from the interface between 30 and 70% Percoll (GE, Pittsburgh, PA, USA). After extensive washing with PBS, cells were collected and subjected to flow cytometry analysis (BD FACSVerse).

### In vitro Th1 and Th17 differentiation

Splenocytes (3 × 10^6^ cells/well) harvested from the spleen of C57BL/6 mice were stimulated with pre-coated anti-mouse CD3 antibody (3 μg/ml, clone: 145-2C11, BioLegend, San Diego, CA) and soluble anti-mouse CD28 antibody (2 μg/ml, clone: 37.51, BioLegend) in the presence of IL-12 (10 ng/ml, BioLegend) for Th1 differentiation or IL-6 (20 ng/ml, BioLegend), TGFβ (10 ng/ml, BioLegend), and anti-mouse IFNγ antibody (5 μg/ml, clone: R4-6A2, BioLegend) for Th17 differentiation. T cells were treated with vehicle or 4-EG 200 μM during Th1 and Th17 differentiation. Forty-eight and 72 hours later, cells were then collected and subjected to flow cytometry analysis for intracellular cytokine expression.

### Flow cytometry analysis for intracellular cytokine and surface marker expression

For intracellular cytokine analysis, mononuclear cells isolated from the brain and spinal cord of C57BL/6 EAE mice, splenocytes harvested from C57BL/6 EAE mice, and naive splenocytes differentiated into Th1 and Th17 cells in vitro were stimulated with phorbol myristate acetate (50 ng/ml, MilliporeSigma) and ionomycin (750 ng/ml, MilliporeSigma) in the presence of Brefeldin A solution (1 μl/ml, BioLegend). After 5 hours of incubation, cells were then harvested and stained with APC-conjugated anti-mouse CD4 (Clone: RM4-5, BioLegend) in the presence of 7-aminoactinomycin D (7-AAD) (BioLegend). Following wash, cells were fixed and then permeabilized followed by intracellular staining with PE/Cy7-conjugated anti-mouse IFNγ (clone: XMG1.2, BioLegend) and PE-conjugated anti-mouse GM-CSF (Clone: MP1-22E9, BioLegend). In addition, cells were stained with Alexa Fluor 488-conjugated anti-mouse CD4 (clone: RM4-5, BioLegend) in the presence of 7-AAD followed by intracellular staining with APC-conjugated anti-mouse IL-17 (clone: TC11-18H10.1, BioLegend). The intracellular expression of IFNγ, IL-17, and GM-CSF in CD4^+^ cells was then determined by flow cytometry analysis, and the flow gating strategies are shown in Fig. S1. For MG surface marker analysis, mononuclear cells isolated from the brain and spinal cord of C57BL/6 EAE mice were stained with APC-conjugated anti-mouse CD11b (clone: M1/70, BioLegend), Alexa Fluor 488-conjugated anti-mouse CD45 (clone: 30-F11, BioLegend), PE/Cy7-conjugated anti-mouse CD80 (clone: 16-10A1, BioLegend), and PE-conjugated anti-mouse CD86 (clone: GL-1, BioLegend). CD80 and CD86 expression on CD45^int^CD11b^+^ MG were then determined by flow cytometry analysis.

### Histopathology and immunohistochemistry (IHC) staining

The L4-L6 segments of the spinal cords harvested from C57BL/6 EAE mice were fixed with 4% paraformaldehyde (MilliporeSigma). For histopathology staining, paraffin-embedded spinal cord sections (8-μm-thick) were subjected to hematoxylin and eosin (H&E) staining, and H&E images were captured with a fluorescence microscope (×10 and ×20, BX53, Olympus). The areas of cell infiltration were determined based on the observation of stained cells near the edge of the spinal cords, and the total areas of cell infiltration were then quantified by ImageJ. For IHC staining, the fixed L4–L6 segments were subjected to sucrose dehydration and then embedded in OCT followed by cryosections. The sections were blocked with 5% goat serum and then stained with anti-MMP9 antibodies (1:100, Cat# 10375-2-AP, Proteintech) at 4 °C overnight. After rinsing with PBS, the sections were stained with secondary antibodies (Alexa Fluor 594 goat anti-rabbit secondary antibody, 1:1000, Invitrogen, Waltham, MA, USA) at room temperature for 2h. After wash, the sections were further stained with Alexa Fluor 488 anti-Iba1 antibodies (1:100, Abcam, EPR16588, Cambridge, UK) at room temperature for 2h followed by coverslipping with ProLong Gold antifade mountant containing DAPI (Invitrogen). Immunofluorescence images of spinal cord tissues were captured with a fluorescence microscope (×20, BX53, Olympus). The slides stained with only secondary antibodies were served as negative controls. Iba1^+^ cells were determined based on the cells with morphology of large cell body and short dendrites, indicating stages 3 to 5 of MG activation [19]. To determine MMP9 fluorescence intensity, negative controls were used to set up the color threshold of MMP9 fluorescence signals, and the mean of MMP9 fluorescence signals (fluorescence intensity) detected on the slides of spinal cord samples prepared from vehicle- and 4-EG-treated EAE mice was calculated by ImageJ.

### Evans blue BBB permeability assay

The BBB permeability was determined based on the leakage of Evans blue as previously described [15]. C57BL/6 EAE mice were i.v. administered with 4 ml/kg 2% (w/v) Evans blue dye dissolved with saline into the lateral tail vein. Two hours after injection, mice were anesthetized and perfused with PBS. The brain and spinal cord were harvested and subjected to imaging to evaluate Evans blue leakage. The tissues were then weighted and homogenized in 50% trichloroacetic acid (TCA) solution. After overnight incubation at 4 °C, the samples were then centrifuged, and the supernatants were extracted and diluted with 95% ethanol in the ratio of 1:3. The amount of Evans blue in the brain and spinal cord tissues was then measured with excitation at 540/25 nm and emission at 640/40 nm by the Synergy^TM^ HT microplate reader (BioTek, Winooski, VT, USA).

### Western blot analysis

Spinal cord and brain tissues harvested from C57BL/6 EAE mice were lysed in radioimmunoprecipitation assay (RIPA) buffer containing 50 mM Tris-HCl (pH8.0), 150 mM NaCl, 1% NP-40, 0.5% sodium deoxycholate, 1 mM PMSF, and 1× protease inhibitor cocktail with 0.3% SDS. Following tissue homogenization, the concentrations of tissue proteins were measured by using the Pierce™ BCA Protein Assay Kit (Thermo Fisher Scientific). The lysate samples were then subjected to electrophoresis and transferred onto the polyvinylidene difluoride membranes (MilliporeSigma). The membranes were then incubated with primary antibodies of MMP9 (1:1000, clone: L51/82, BioLegend), MMP3 (1:1000, clone: M4405F10, BioLegend), Nrf2 (1:100, Cat# 16396-I-AP, Proteintech), HO-1 (1:2000, Cat# 10701-I-AP, Proteintech), or β-actin (BD Biosciences) overnight followed by incubation with horseradish peroxidase (HRP)-conjugated secondary antibodies (BD Biosciences) for 1 hour. Immunoreactivities were then visualized by Immobilon™ Western Chemiluminescent HRP Substrate (MilliporeSigma). The intensity of designated protein bands was densitometrically quantified and then normalized with β-actin by using the Image Pro-Plus 4.5 (Media Cybernetics, Rockville, MD, USA).

### Cell culture

Primary MG were generated from P1-P2 neonatal mice as previously described [20]. Briefly, cerebral cortical cells were harvested from P1-P2 neonatal mice and then plated in 75-cm^2^ culture flasks in a complete medium (DMEM/F12 supplemented with 10% FBS). At day 4 and 8 after plating, the medium was removed and replenished with a complete medium containing 10 ng/ml GM-CSF. The flasks were then shaken at 37 °C for 30 minutes at day 12, and MG were harvested from supernatants for experiments. The mouse microglial cell line BV2 cells were grown in 25-cm^2^ flasks. After cells were grown to confluence, cells were trypsinized and seeded on tissue culture plates followed by indicated stimulations. Primary macrophages were generated from bone marrow cells as previously described [21]. Briefly, bone marrow cells were cultured with a complete RPMI 1640 medium containing 10 ng/ml M-CSF. Cells were replenished with a fresh medium containing 10 ng/ml M-CSF at day 3 and collected at day 7 for experiments.

### Quantitative PCR

The expression of *Ho-1*, *NQO1*, and *Gclc* was detected by SYBR green-based quantitative PCR (Q-PCR). The primers used were as follows: *Ho-1*, sense 5′-GCTGGTGATGGCTTCCTTGT-3′ and antisense 5′-ACTGGGTTCTGCTTGTTGCG-3′; *Nqo1*, sense 5′-GGCCCATTCAGAGAAGACAT-3′ and antisense 5′-TTCGAGTACCTCCCATCCTC-3′; *Gclc*, sense 5′-CCTACGGAGGAA CGATGTCT-3′ and antisense 5′-GGAATGAAGTGATGGTGCAG-3′; and *β-actin*, sense 5′-TCCACCACCACAGCTGAGAGG-3′ and antisense 5′-CAGCTTCTCTTTGATGTCACGC-3′. Q-PCR was performed using the Applied Biosystems StepOnePlus^TM^ Real-Time PCR System. The levels of gene expression were determined by the cycle threshold (Ct) values followed by quantification using standard curves. The results were then normalized to β-actin from the same samples to obtain relative changes of gene expression.

### Statistical analysis

All results were presented as mean ± SEM. For samples that passed the Shapiro-Wilk normality test, comparisons between two groups were performed by unpaired *t* test, whereas comparisons among multiple groups were performed by one-way ANOVA followed by Tukey post hoc test. Two-way ANOVA test was used for statistical analysis of EAE clinical scores. For samples that did not pass the normality test, comparisons between two groups were performed by Mann-Whitney *U* test. Statistical analysis was performed by using the GraphPad Prism 8 software, and statistical significance was determined as *p* values ≤ 0.05.

## Results

### 4-EG ameliorates disease severity in chronic EAE and alleviates disease progression in relapsing-remitting EAE

**Fig. 1:**
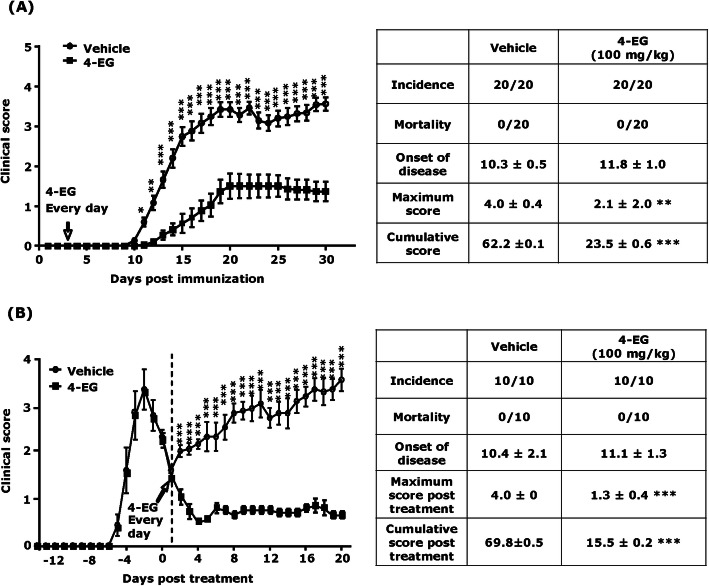
4-EG ameliorates disease severity in chronic EAE and alleviates disease progression in relapsing-remitting EAE. **a** C57BL/6 mice were immunized with MOG_35–55_ and then i.p. injected with vehicle or 4-EG 100 mg/kg (*n*=20/group) daily starting from day 3 post-immunization. The clinical score of EAE animals was followed for 30 days. **b** SJL/J mice were immunized with PLP_139–151_ to induce EAE. Following the first remission and reaching disease score less than 1.5, SJL/J EAE mice were i.p. injected with vehicle or 4-EG 100 mg/kg (*n*=10/group) daily. The clinical score of EAE mice was monitored for 20 days. Statistical significance of EAE clinical score was determined as *p<0.05, **p<0.01, and ***p<0.001 by two-way ANOVA test. EAE incidence, mortality, onset of disease, maximum score, and cumulative score of C57BL/6 and SJL/J EAE were also assessed. Statistical significance was determined as **p<0.01 and ***p<0.001 by Mann-Whitney *U* test

To explore whether 4-EG offers protection against EAE, we evaluated the effects of 4-EG on the amelioration of disease severity in C57BL/6 chronic EAE models. MOG_35–55_-immunized C57BL/6 mice were i.p. administered with vehicle or different doses of 4-EG (50, 100, and 200 mg/kg) daily starting from day 3 post-immunization, and the clinical scores of vehicle- and 4-EG-treated EAE mice were monitored for 30 days. We observed 4-EG 50 mg/kg offered little protection in EAE. However, 4-EG 200 mg/kg exerted a toxic effect that resulted in 100% mortality in EAE mice. We found that 4-EG 100 mg/kg offered optimal protection in EAE and did not cause any toxicity in EAE mice (Fig. S2). Thus, the dose of 100 mg/kg 4-EG was selected and used throughout the studies. Using the dose of 100 mg/kg 4-EG for the treatment of EAE, we found that although vehicle- and 4-EG-treated EAE mice had a comparable outcome with regard to the onset of disease, 4-EG treatment ameliorated disease severity and significantly reduced both maximum and cumulative disease scores compared to vehicle treatment in EAE mice (maximum score, 4-EG 2.1±2.0 vs. vehicle 4.0±0.4; cumulative disease score, 4-EG 23.5±0.6 vs. vehicle 62.2±0.1) (Fig. 1a).

We then evaluated whether 4-EG exerted a therapeutic potential in the relapsing-remitting EAE model. SJL/J mice were immunized with PLP_139–151_ to induce relapsing-remitting EAE. After EAE mice entered the first remitting phase (disease score ≤1.5), EAE mice were then administered with vehicle or 4-EG, and the disease scores of EAE mice were monitored for additional 20 days. Our results showed 4-EG treatment not only ameliorated disease severity but also prevented disease relapse in SJL/J EAE mice. In contrast, vehicle-treated SJL/J EAE mice further developed relapses accompanied with augmented disease scores. Our results showed that the maximum and cumulative scores post-treatment were 1.3±0.4 and 15.5±0.2 for 4-EG-treated EAE mice and 4.0±0 and 69.8±0.5 for vehicle-treated EAE controls, respectively (Fig. 1b). Collectively, our results demonstrate that 4-EG ameliorates disease severity in chronic EAE and attenuates disease progression in relapsing-remitting EAE, suggesting that 4-EG exerts protective and therapeutic effects in EAE.

### 4-EG suppresses encephalitogenic Th1 and Th17 infiltration of the CNS in EAE

**Fig. 2:**
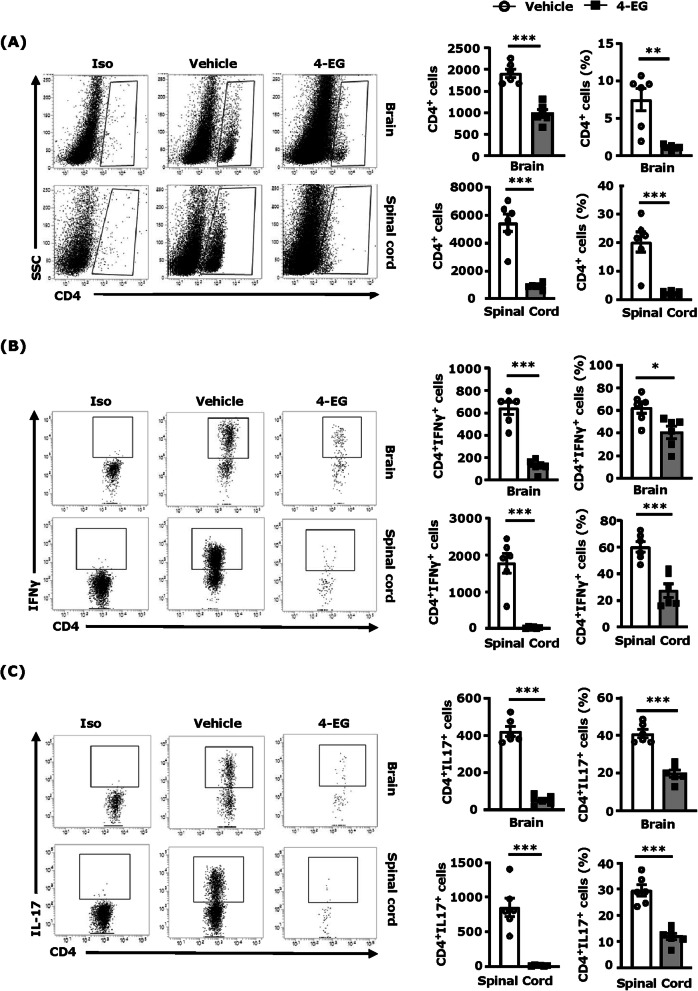
4-EG suppresses encephalitogenic Th1 and Th17 infiltration of the CNS in EAE. At day 11–13 post-immunization, mononuclear cells were isolated from the brain and spinal cord of vehicle- and 4-EG (100 mg/kg)-treated C57BL/6 EAE mice (*n*=6/group), and the isolated mononuclear cells were then subjected to surface staining of CD4 followed by intracellular staining of IFNγ and IL-17. **a**–**c** The frequency determined by the percentages of CD4^+^ T cells in total acquired cells, and the percentages of IFNγ- or IL-17-expressing CD4^+^ T cells in total CD4^+^ T cells were analyzed in the brain and spinal cord of vehicle- and 4-EG-treated EAE mice by flow cytometry analysis. **a**–**c** The number of CD4^+^ T cells, CD4^+^ IFNγ^+^ T cells, and CD4^+^ IL-17^+^ T cells in the brain and spinal cord of vehicle- and 4-EG-treated EAE mice was also determined. One representative flow result of each condition is shown. Isotype controls (Iso) were used as a negative control to determine cells positive for the surface expression of CD4 **a** or CD4^+^ T cells positive for the intracellular expression of IFNγ or IL-17 **b**, **c**. Data are representative of three independent experiments (*n*=4–6/group per experiment). Statistical significance was determined as *p<0.05, **p<0.01, and ***p<0.001 by unpaired *t* test

Given that the CNS infiltration of encephalitogenic Th1 and Th17 exerts the main pathogenic effect on the induction of EAE, we evaluated whether 4-EG treatment modulated pathogenic CD4^+^ T cell infiltration of the CNS in C57BL/6 EAE mice. Mononuclear cells isolated from the brain and spinal cord of vehicle- and 4-EG-treated EAE mice were subjected to flow cytometry analysis to determine the number and frequency of the CNS infiltrating CD4^+^ T cells as well as IFNγ- and IL-17-expressing CD 4^+^ T cells in the brain and spinal cord of EAE. Our results showed that a large number of CD4^+^ T cells were observed in the brain and spinal cord of vehicle-treated EAE mice. In contrast, the number of CD4^+^ T cells was significantly reduced in the brain and spinal cord of 4-EG-treated EAE mice (Fig. 2a). Similarly, we observed that the frequency (%) of CD4^+^ T cells in the brain and spinal cord was also significantly lower in 4-EG-treated EAE mice than vehicle-treated EAE controls (Fig. 2a). We further analyzed Th1 and Th17 cells in the CNS of EAE mice and found that the number and frequency of IFNγ- and IL-17-expressing CD 4^+^ T cells in the brain and spinal cord were significantly decreased in 4-EG-treated EAE mice compared to vehicle-treated EAE controls (Fig. 2b, c). Altogether, these results demonstrate that 4-EG suppresses Th1 and Th17 infiltration of the CNS that may contribute to its protective effects in EAE.

### 4-EG inhibits Th1 and Th17 differentiation in vivo and in vitro

**Fig. 3:**
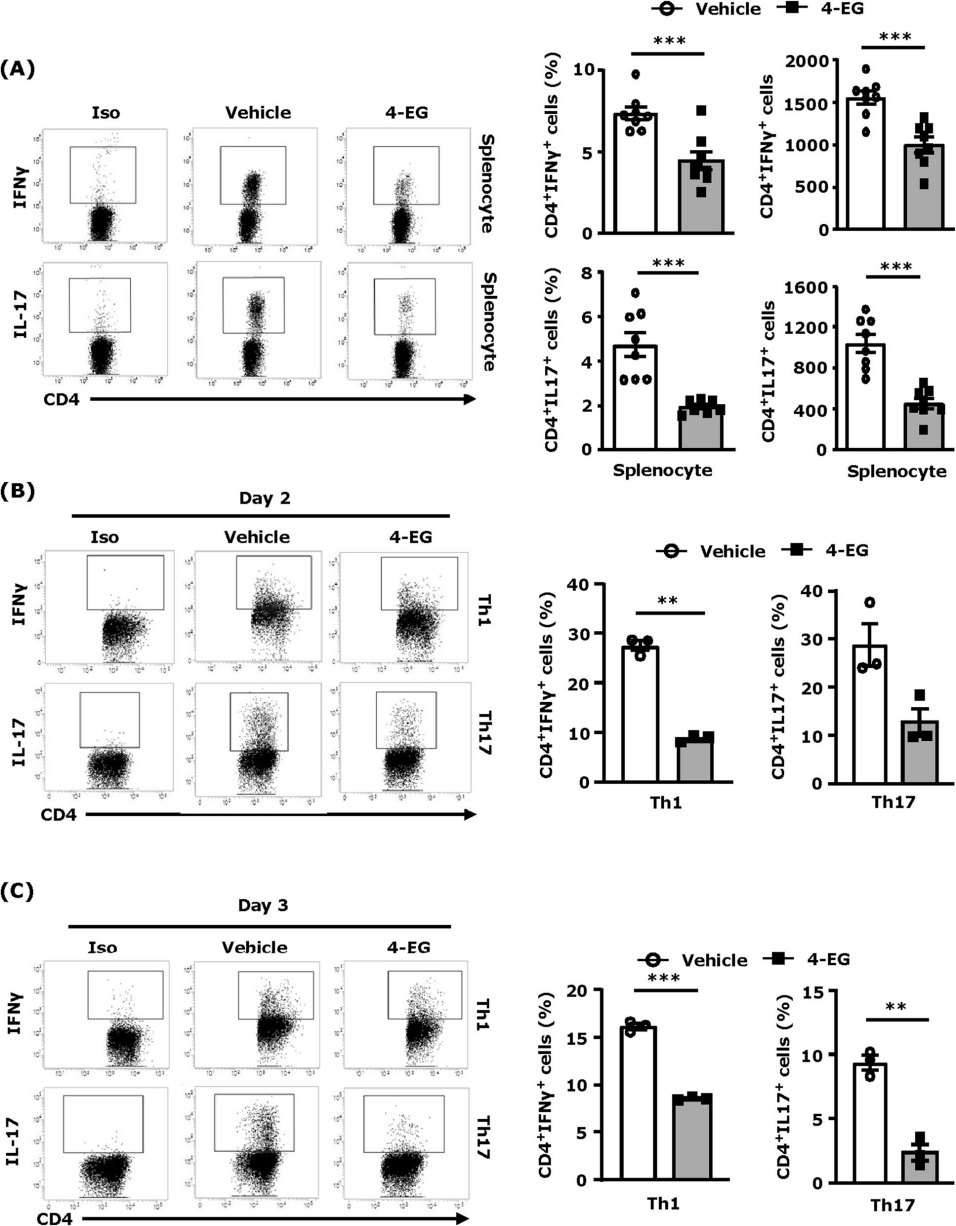
4-EG inhibits Th1 and Th17 differentiation in vivo and in vitro. **a** Splenocytes harvested from vehicle- and 4-EG (100 mg/kg)-treated C57BL/6 EAE (*n*=8/group) at day 8 post-immunization were subjected to flow cytometry analysis to determine the frequency and number of IFNγ- and IL-17-expressing CD4^+^ T cells. **b**, **c** Naive splenocytes were polarized into Th1 and Th17 conditions in the presence or absence of 4-EG (200 μM). Forty-eight and 72 hours after incubation, cells were collected and subjected to flow cytometry analysis to assess the frequency of intracellular expression of IFNγ or IL-17 in CD4^+^ T cells. Data are representative of four independent experiments (*n*=2–3/technique replicates per experiment). Isotype controls (Iso) were used as a negative control to determine CD4^+^ cells positive for intracellular expression of IFNγ or IL-17. Statistical significance was determined as *p<0.05, **p<0.01, and ***p<0.001 by unpaired *t* test

To elucidate whether 4-EG modulates CD4^+^ T cell differentiation in the periphery that results in repressed Th1 and Th17 infiltration of the CNS in EAE, splenocytes were harvested from vehicle- and 4-EG-treated EAE mice at day 8 post-immunization followed by flow cytometry analysis to determine the level of IFNγ- and IL-17-expressing CD4^+^ T cells. Our results showed that the frequency and number of IFNγ- and IL17-expressing CD4^+^ T cells were significantly lower in the spleen of 4-EG-treated EAE mice compared to that of vehicle-treated EAE controls (Fig. 3a), indicating 4-EG suppresses Th1 and Th17 differentiation in the periphery of EAE.

To further confirm the suppressive effect of 4-EG on Th1 and Th17 differentiation, splenocytes harvested from naive mice were differentiated into Th1 or Th17 cells in the presence or absence of 200 μM 4-EG in vitro followed by flow cytometry analysis to determine the intracellular expression of IFNγ and IL-17 in CD4^+^ T cells. Our results showed that 4-EG strongly inhibited Th1 and Th17 differentiation in vitro because the intracellular expression of IFNγ and IL-17 in CD4^+^ T cells was suppressed at day 2 and day 3 post-treatment (Fig. 3b, c). Although only live CD4^+^ T cells were gated to assess intracellular cytokine expression (Fig. S1C), we thought to evaluate whether 4-EG exerts toxicity in T cells. Our results showed that the cell viability of vehicle- and 200 μM 4-EG-treated Th1 and Th17 cells was comparable at day 2 post-treatment (Fig. S3). In addition, we observed vehicle- and 200 μM 4-EG-treated Th1 cells displayed a similar viability at day 3 post-treatment (Fig. S3A). Although 4-EG decreased cell viability in Th17 cells about 25% at day 3 post-treatment (Fig. S3B), 4-EG exhibited a strong suppressive effect on Th17 differentiation that led to a more than 4-fold decrease in Th17 cells compared to vehicle (Fig. 3c). These results suggest that 4-EG-suppressed Th1 and Th17 differentiation is not due to its toxicity. Taken altogether, our results demonstrate that 4-EG suppresses pathogenic Th1 and Th17 differentiation in vitro and in vivo, and that may then result in decreased Th1 and Th17 infiltration in the brain and spinal cord of 4-EG-treated EAE mice.

### 4-EG represses MG activation in EAE

**Fig. 4:**
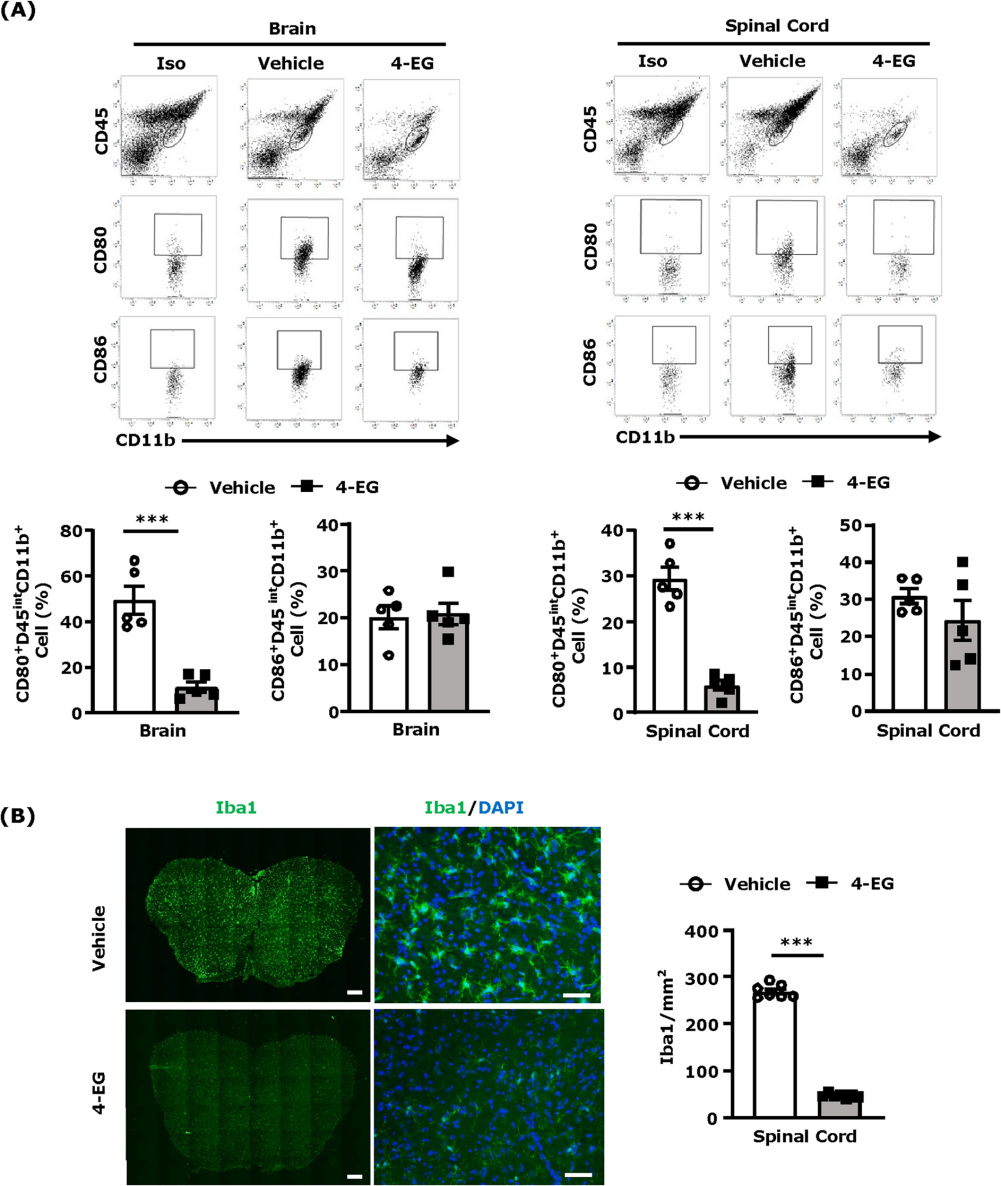
4-EG represses MG activation in EAE. **a** At day 12–13 post-immunization, mononuclear cells were isolated from the brain and spinal cord of vehicle- and 4-EG (100 mg/kg)-treated C57BL/6 EAE mice followed by flow cytometry analysis to determine CD80 and CD86 expression on CD45^int^CD11b^+^ MG (*n*=5/group). Isotype controls (Iso) were used as a negative control to determine MG positive for CD80 or CD86 expression. Data are representative of two independent experiments (*n*=5/group per experiment). **b** The lumbar regions of spinal cord tissues were collected from vehicle- and 4-EG-treated C57BL/6 EAE mice (*n*=7/group) followed by immunofluorescence analysis to determine cells positive for Iba1 expression. The number of Iba1^+^ cells was calculated and quantified under ×20 magnification. Scale bars, 200 μm (×10, left image), 50 μm (×20, right image). Statistical significance was determined as ***p<0.001 by unpaired *t* test

MG activation leads to increased production of pro-inflammatory cytokines and chemokines that induce neuroinflammation and promote the infiltration of inflammatory immune cells into the CNS of EAE [5]. We hypothesized that 4-EG might suppress MG activation to attenuate neuroinflammation and repress inflammatory immune cell infiltration of the CNS in EAE. To elucidate that, we isolated mononuclear cells from the brain and spinal cord of vehicle- and 4-EG-treated EAE mice and evaluated MG activation based on their expression of maturation markers, CD80 and CD86 [22–24]. Although a similar level of CD86^+^ MG was observed in the CNS of vehicle- and 4-EG-treated EAE mice, our results showed that the number of CD80^+^ MG was profoundly reduced in the brain and spinal cord of 4-EG-treated EAE mice compared to those of vehicle-treated EAE controls (Fig. 4a). To further confirm our observation of 4-EG suppression of MG activation in EAE, we analyzed the level of Iba1^+^ cells in the spinal cord of vehicle- and 4-EG-treated EAE mice. Our results showed that there was a substantial reduction of Iba1^+^ cells in the spinal cord of 4-EG-treated EAE mice compared to that of vehicle-treated EAE controls (Fig. 4b). Altogether, these results illustrate that 4-EG suppresses MG activation to lessen neuroinflammation in EAE.

### 4-EG mitigates BBB disruption, spinal cord pathology, and MMP3/9 expression in EAE

**Fig. 5:**
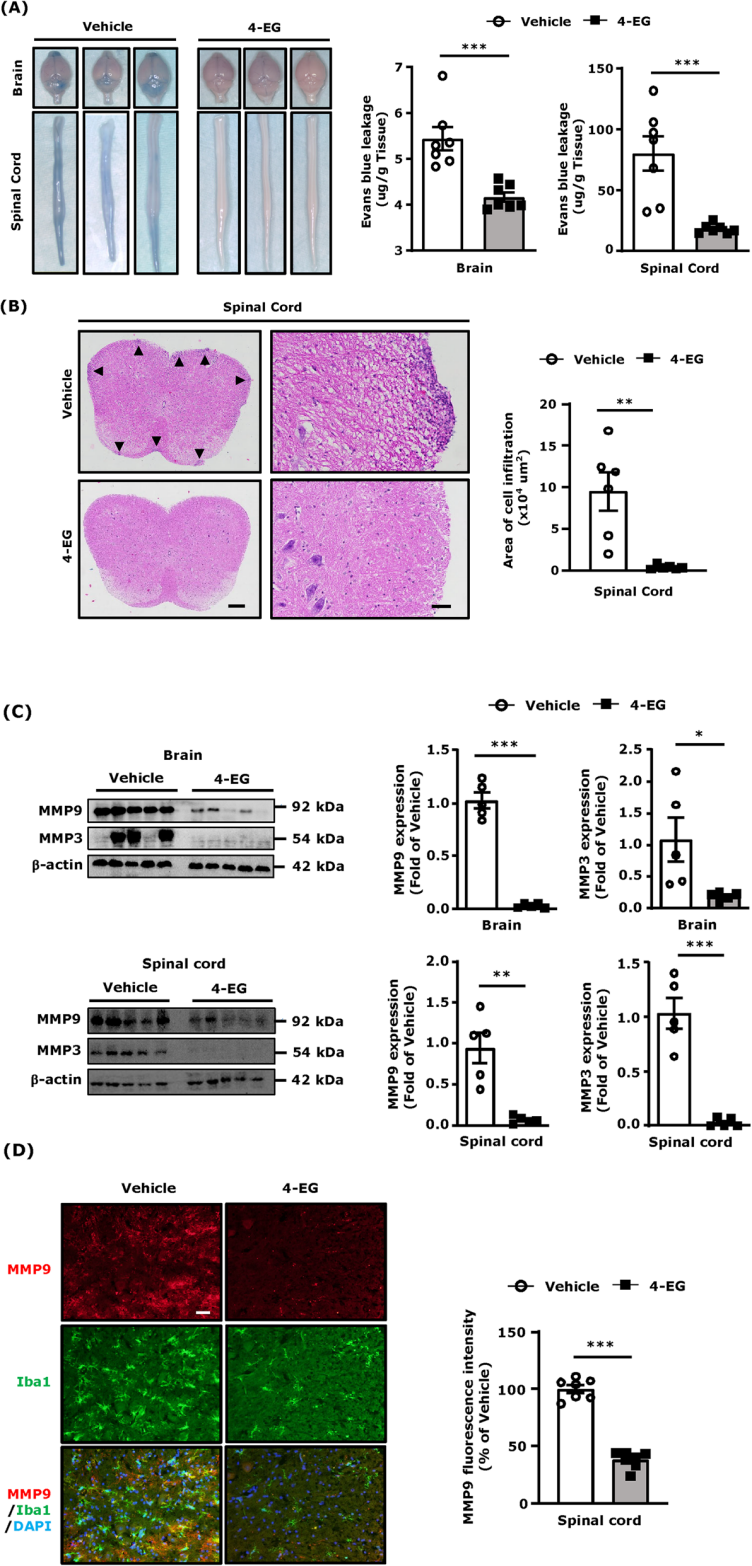
4-EG mitigates BBB disruption, spinal cord pathology, and MMP3/9 expression in EAE. **a** At day 12–13 post-immunization, vehicle- and 4-EG (100 mg/kg)-treated C57BL/6 EAE mice were subjected to i.v. injection of Evans blue. Two hours after Evans blue injection, EAE mice were sacrificed, and the brains and spinal cords were harvested to analyze Evans blue leakage. Representative brain and spinal cord images of three vehicle- and 4-EG-treated EAE are shown, and the amount of Evans blue leakage in the brain and spinal cord tissues was also quantified (*n*=7/group). **b** The lumbar regions of the spinal cord harvested from vehicle- and 4-EG-treated C57BL/6 EAE mice were subjected to H&E staining to determine peripheral immune cell infiltration. Representative H&E images of the coronal spinal cord sections are shown. Scale bars, 200 μm (×10, left image), 50 μm (×20, right image). The areas of cell infiltration were quantified (*n*=6/group). **c** The brain and spinal cord tissues were harvested from vehicle- and 4-EG-treated C57BL/6 EAE mice (*n*=5/group) and then subjected to western blot analysis to measure MMP3 and MMP9 expression. Data are representative of two independent experiments (*n*=5/group per experiment). **d** The lumbar regions of the spinal cord from vehicle- and 4-EG-treated C57BL/6 EAE mice were subjected to IHC to determine MMP9 and Iba1 expression. Representative images of MMP9 and Iba1 expression are shown. The fluorescence intensity of MMP9 was also quantified (*n*=7/group). Scale bar, 50 μm (×20). Statistical significance was determined as *p<0.05, **p<0.01, and ***p<0.001 by unpaired *t* test

BBB disruption is considered to be the pathological hallmark of MS/EAE [25]. To explore the effects of 4-EG on BBB disruption in EAE, we examined the level of BBB leakage in vehicle- and 4-EG-treated EAE mice. At day 12–13 post-immunization, vehicle- and 4-EG-treated EAE mice were i.v. administered with Evans blue, and the level of Evans blue leakage in the brain and spinal cord tissues was then determined. Our results showed a clear image of Evans blue leakage in the brain and spinal cord of vehicle-treated EAE mice. In contrast, Evans blue leakage was not clearly observed in the CNS of 4-EG-treated EAE mice (Fig. 5a, left). We then quantified the amount of Evans blue leakage in the CNS, and our results showed that the amount of Evans blue in the brain and spinal cord was significantly lower in 4-EG-treated EAE mice compared to vehicle-treated EAE controls (Fig. 5a, right). The histopathological features of the spinal cord in EAE were also assessed to determine whether increased BBB leakage positively correlates with elevated cell infiltrates in the CNS. We observed a large number of infiltrating immune cells in the spinal cord of vehicle-treated EAE mice. In contrast, only few infiltrating immune cells were observed in the spinal cord of 4-EG-treated EAE mice (Fig. 5b).

Previous studies demonstrated that MMP3 and MMP9 contribute to the pathology of EAE/MS by aggravating BBB disruption [26, 27]. To elucidate whether the protective effect of 4-EG on maintaining BBB integrity in EAE was due to modulation of MMP3 and MMP9 expression, the brain and spinal cord tissues harvested from vehicle- and 4-EG-treated EAE mice were subjected to western blot analysis to determine MMP3 and MMP9 expression. Our results showed that both MMP3 and MMP9 were highly upregulated in the brain and spinal cord of vehicle-treated EAE mice (Fig. 5c). In contrast, the expression of MMP3 and MMP9 was largely suppressed in the brain and spinal cord of 4-EG-treated EAE mice (Fig. 5c). Furthermore, immunofluorescence analysis showed that MMP9 was highly expressed in the lumbar region of the spinal cord of vehicle-treated EAE mice and displayed as a secreted form as well as an intracellular form that was co-localized with Iba1^+^ cells. On the contrary, MMP9 expression was strongly suppressed in the spinal cord of 4-EG-treated EAE mice (Fig. 5d). Taken altogether, our results demonstrate that 4-EG mitigates EAE-induced BBB disruption that may be mediated through the anti-inflammatory effect of 4-EG on the suppression of MMP3 and MMP9 production.

### 4-EG suppresses GM-CSF expression in vivo and in vitro

**Fig. 6:**
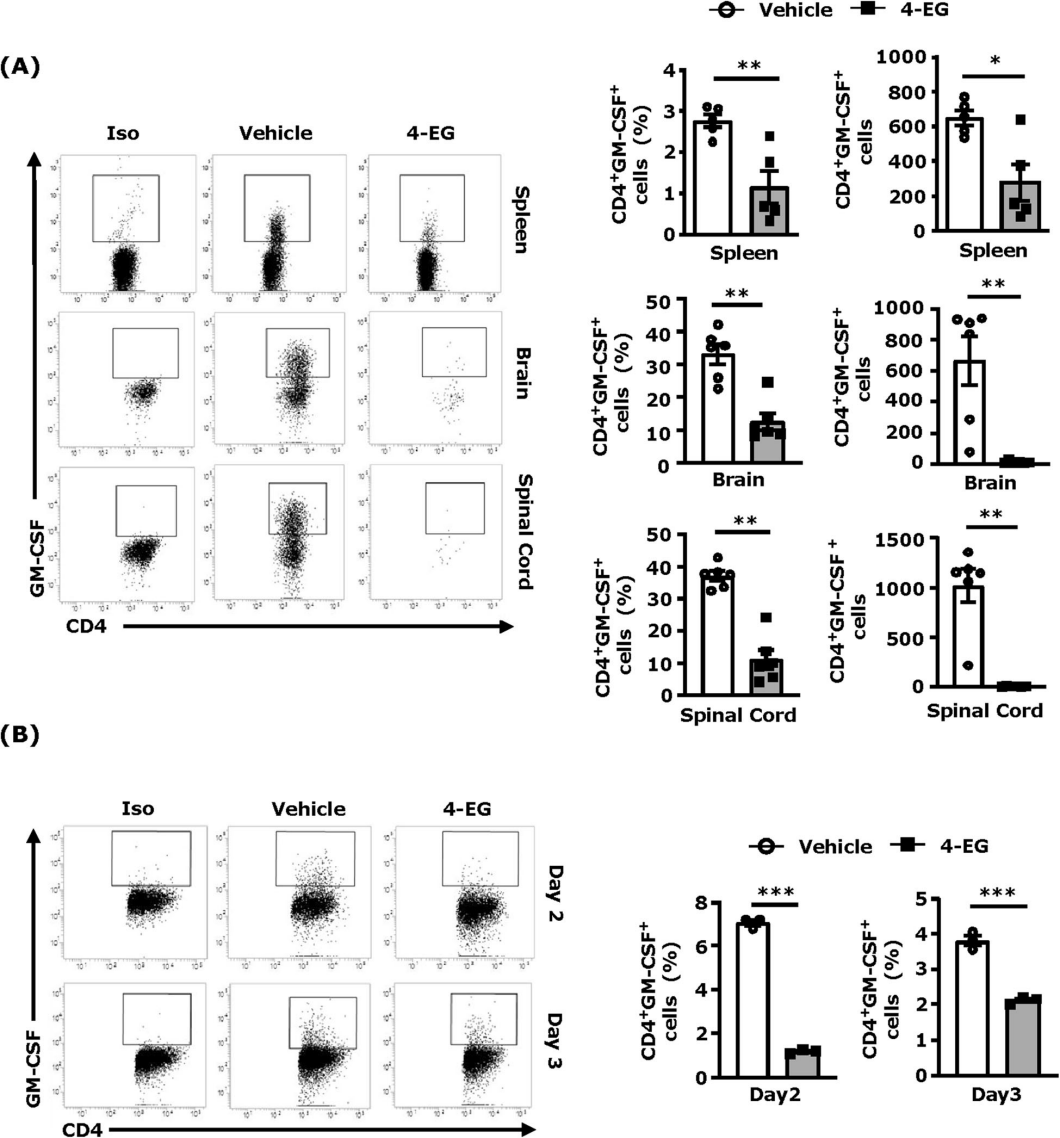
4-EG suppresses GM-CSF expression in vivo and in vitro. **a** Splenocytes were harvested from vehicle- and 4-EG (100 mg/kg)-treated C57BL/6 EAE mice at day 8 post-immunization (*n*=5/group), and mononuclear cells were isolated from the brain and spinal cord of vehicle- and 4-EG-treated C57BL/6 EAE mice at day 11–13 post-immunization (n=6/group). Splenocytes and the isolated mononuclear cells were then subjected to surface staining of CD4 followed by intracellular staining of GM-CSF. The frequency and number of GM-CSF-expressing CD4^+^ T cells in the spleen, brain, and spinal cord were analyzed by flow cytometry analysis. Isotype controls (Iso) were used as a negative control to determine cells positive for the intracellular expression of GM-CSF. **b** Naive splenocytes were polarized into Th1 conditions in the presence or absence of 4-EG (200 μM). Forty-eight and 72 hours after incubation, cells were collected and subjected to flow cytometry analysis to assess the frequency of intracellular expression of GM-CSF in CD4^+^ T cells. Iso were used as a negative control to determine CD4^+^ cells positive for the intracellular expression of GM-CSF. Data are representative of four independent experiments (*n*=2–3/technique replicates per experiment). Statistical significance was determined as *p<0.05, **p<0.01, and ***p<0.001 by Mann-Whitney *U* test

GM-CSF has been reported to play an essential role in the development and progression of EAE [3]. Although various cell types produce GM-CSF that include T cells, monocytes/macrophages, and endothelial cells [28], GM-CSF-producing CD4^+^ T cells have been shown to be pivotal for EAE development [3]. To explore whether 4-EG affects GM-CSF production in EAE, splenocytes and mononuclear cells isolated from the brain and spinal cord of vehicle- and 4-EG-treated EAE mice were subjected to flow cytometry analysis to determine the frequency and number of GM-CSF-expressingproducing CD4^+^ T cells. Our results showed that 4-EG treatment decreased the frequency and number of GM-CSF-expressing CD4^+^ T cells in the spleen of EAE mice, demonstrating that 4-EG inhibits the differentiation of GM-CSF-expressing CD4^+^ T cells in the periphery of EAE (Fig. 6a, top). Consequently, we observed a significant reduction of GM-CSF^+^ CD4^+^ T cells in the brain and spinal cord of 4-EG-treated EAE mice compared to those of vehicle-treated EAE controls (Fig. 6a, middle and bottom).

To investigate whether 4-EG exerts a direct effect on the suppression of GM-CSF production in CD4^+^ T cells, splenocytes were harvested and differentiated into Th1 cells in the presence or absence of 4-EG, and the intracellular expression of GM-CSF in Th1 cells was then analyzed. Our results showed that the frequency of GM-CSF-expressing CD4^+^ T cells was significantly decreased in 4-EG-treated Th1 cells compared to vehicle-treated Th1 controls (Fig. 6b). Collectively, these results demonstrate that 4-EG suppresses GM-CSF production in CD4^+^ T cells in vitro and in vivo.

### 4-EG induces HO-1 expression in vivo and in vitro

**Fig. 7:**
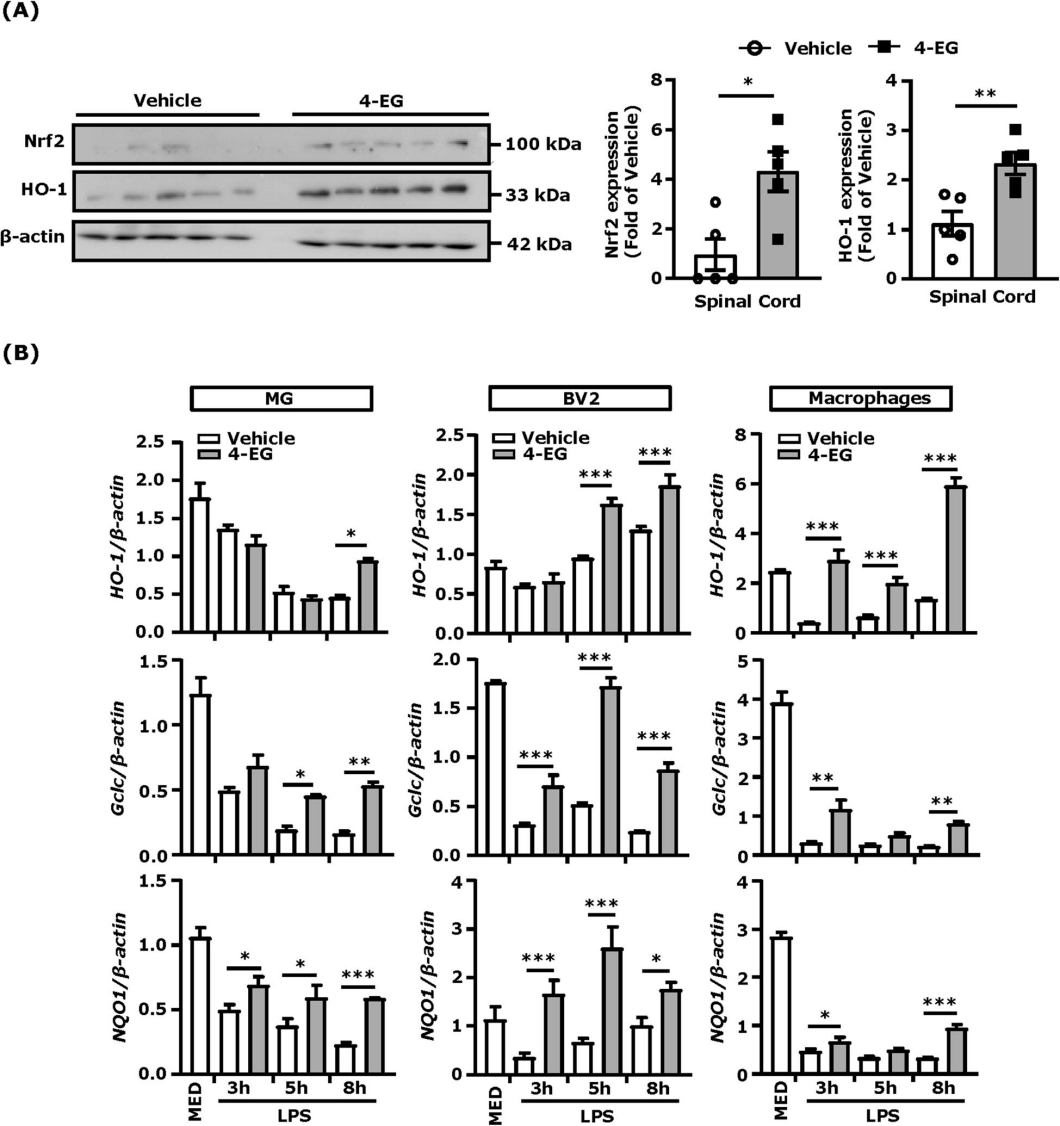
4-EG induces HO-1 expression in vivo and in vitro. **a** The spinal cords harvested from vehicle- and 4-EG (100 mg/kg)-treated C57BL/6 EAE mice (*n*=5/group) at day 12–13 post-immunization were subjected to western blot analysis to measure Nrf2 and HO-1 expression. The level of Nrf2 and HO-1 expression was quantified and then normalized with β-actin, and the fold changes compared with vehicle are shown. *p<0.05 and **p<0.01 by Mann-Whitney *U* test. Data are representative of two independent experiments (*n*=5/group per experiment). **b** MG, BV2 cells, and macrophages were pretreated with 4-EG 400 μM for 1 hour followed by LPS 100 ng/ml stimulation for a time course. Cells were then harvested and subjected to Q-PCR analysis for HO-1, Gclc, and NQO1 mRNA expression. Data are representative of 3–4 independent experiments (*n*=3/technique replicates per experiment). Statistical significance was determined as *p<0.05, **p<0.01, and ***p<0.001 by one-way AVONA test

Induction of Nrf2/HO-1 pathway in EAE has been shown to offer protection against EAE [29–31]. Previously, studies showed that 4-EG induced HO-1 expression in THP-1 cells [8]. To elucidate whether 4-EG induces Nrf2/HO-1 pathway to offer protection against EAE, the level of Nrf2 and HO-1 expression was measured in the spinal cord of vehicle- and 4-EG treated-EAE mice. We observed increased expression of Nrf2 and HO-1 in the spinal cord of 4-EG-treated EAE mice compared to that of vehicle-treated EAE controls (Fig. 7a).

Nrf2 activation contributes to the induction of phase II detoxification and anti-oxidant enzymes, including HO-1, Gclc, and NQO1 [32]. To confirm the effect of 4-EG on the induction of the Nrf2/HO-1 pathway, primary MG, BV2 cells, and primary macrophages were activated with LPS in the presence or absence of 4-EG in a time-dependent manner, and the expression of HO-1, Gclc, and NQO1 was then measured. Our results showed that 4-EG induced the expression of all three enzymes in MG, BV2 cells, and macrophages (Fig. 7b). Altogether, these results demonstrate that 4-EG induces HO-1 expression in vitro and in vivo, and 4-EG-induced HO-1 expression in the CNS of EAE may contribute to its protective effect on the amelioration of EAE.

## Discussion

4-EG, a phenolic compound, has been reported to be a potential anti-inflammatory modulator through repressing the activation of NFκB and inflammasome, and inhibiting the production of inflammatory cytokines [8]. However, it remains unknown whether 4-EG exerts anti-inflammatory effects on the modulation of CNS diseases. In this study, we evaluated the therapeutic effect of 4-EG in autoimmune neuroinflammatory disease using the animal model of EAE. We observed that 4-EG treatment ameliorated disease severity and mitigated disease relapse in chronic and relapsing EAE, respectively. In addition, we found 4-EG attenuated MG activation, inhibited Th1/Th17 differentiation in the periphery, suppressed Th1/Th17 infiltration of the CNS, and lessened BBB disruption in EAE. Mechanistic studies revealed that the induction of HO-1 might be responsible for the protective effect of 4-EG in EAE. Thus, we show for the first time that 4-EG confers protection against EAE and could be developed as a novel therapeutic agent for the treatment of autoimmune disease EAE.

Pathogenic immune or inflammatory cells are essential for EAE development and progression after infiltrating into the CNS [33]. Here, we found 4-EG suppressed the differentiation of Th1 and Th17 cells in the spleen of EAE, and that might subsequently repress Th1 and Th17 infiltration of the CNS and attenuate neuroinflammation in EAE. Consequently, 4-EG-treated EAE mice exhibited ameliorated disease severity and mitigated EAE pathology compared to vehicle-treated EAE controls. As the suppression of Th1 and Th17 differentiation was observed in the periphery of 4-EG-treated EAE mice, we thought to determine whether 4-EG-suppressed Th1/Th17 differentiation was due to the direct effect of 4-EG on the inhibition of pathogenic Th1/Th17 differentiation. We found that in vitro culture of splenocytes under Th1 and Th17 differentiation conditions in the presence of 4-EG inhibited the production of IFNγ and IL-17, respectively. These results suggest that 4-EG exerts a direct effect on the suppression of Th1/Th17 differentiation. However, whether 4-EG modulates the production of inflammatory cytokines, such as IL-12 and IL-23, to indirectly suppress Th1 and Th17 differentiation would require further investigations.

MG activation has been reported to be a major modulator in the pathogenesis of autoimmune disorders [34]. Following activation, MG rapidly produce a large amount of pro-inflammatory cytokines and chemokines that promote the recruitment of peripheral immune cells into the CNS, leading to neuroinflammation and subsequent oligodendrocyte death and demyelination in MS/EAE [5]. Because of that, the modulation of MG activation is thought to be a therapeutic strategy for CNS autoimmune diseases. In this study, we demonstrated that 4-EG attenuated MG activation, as we observed the number of CD80^+^ and Iba1^+^ MG was largely decreased in 4-EG-treated EAE mice compared to vehicle-treated EAE controls. In addition, our in vitro studies showed that 4-EG was capable of upregulating phase II detoxification and anti-oxidant enzymes, HO-1, Gclc, and NQO1, in primary MG, and these enzymes have been shown to exert anti-oxidant and anti-inflammatory effects. Altogether, our results suggest that the therapeutic potential of 4-EG in EAE may be in part mediated through its effect on inhibiting MG activation that subsequently attenuates neuroinflammation in EAE.

Recently, it has been shown that GM-CSF plays a pathogenic role in MS [3]. GM-CSF not only exerts an important function in the encephalitogenicity of Th1 and Th17 cells but also promotes proliferation and activation of MG and macrophages, which are essential for the onset of MS/EAE progression [35–37]. Furthermore, GM-CSF can boost the differentiation of M1-like macrophages and induce the production of inflammatory cytokines, such as IL-1β, IL-6, and TNFα, that may then result in the destruction of myelin sheath [38, 39]. Moreover, GM-CSF facilitates the inflammasome processing of IL-1β in monocytes and macrophages that further enhances Th17 expansion and BBB disruption [36, 39]. In our study, we found that CD4^+^ T cells produced GM-CSF in the spleen, brain, and spinal cord of vehicle-treated EAE mice that might further aggravate disease progression. In contrast, 4-EG-treated EAE mice exhibited reduced GM-CSF-expressing CD4^+^ T cells in the periphery as well as in the CNS. These results were further confirmed by our in vitro studies in which GM-CSF-expressing CD4^+^ T cells were also decreased in Th1 cultures when 4-EG was present. Collectively, our findings demonstrate that 4-EG possesses an inhibitory effect on the production of GM-CSF in pathogenic T cells in vivo as well as in vitro.

Studies have confirmed that matrix metalloproteinases, especially MMP3 and MMP9, are dysregulated in various diseases, including MS, that result in elevated BBB disruption and increased leukocyte trafficking [40, 41]. Consistent with previous studies, we found MMP3 and MMP9 were largely produced in the CNS of EAE mice that correlated with augmented BBB disruption with elevated Evans blue leakage in the CNS. In addition, increased cell infiltrates were observed in the brain and spinal cord of EAE. In contrast, 4-EG treatment was able to suppress MMP3 and MMP9 production, mitigate BBB disruption, and repress cell infiltrates in the CNS of EAE. These results illustrate the protective effect of 4-EG on maintaining BBB integrity. Furthermore, previous studies show that GM-CSF exerts a detrimental effect on attenuating the expression of tight junction proteins (TJP), claudin-5 and ZO-1, in HBMECs [33, 42]. As the expression of claudin-5 and ZO-1 is essential for preserving BBB integrity, 4-EG-mediated suppression of GM-CSF may play a role in preventing TJP degradation to preserve BBB’s function. However, further studies are required to demonstrate the potential protective effect of 4-EG on attenuating TJP degradation in EAE.

Finally, we investigated the potential molecular mechanism underlying the protective effect of 4-EG in EAE. Previous studies have demonstrated that the induction of HO-1 confers protection against EAE [29, 43]. For instance, HO-1 inducers, including dimethyl fumarate and 3H-1,2-dithiole-3-thione, have been shown to ameliorate EAE severity [44, 45]. We found that HO-1 was highly induced in the spinal cord of 4-EG-treated EAE mice compared to that of vehicle-treated EAE controls. Since MG and macrophages were reported to express HO-1 [46, 47], we evaluated whether 4-EG promoted HO-1 induction in both MG and macrophages. Indeed, we observed 4-EG induced HO-1 upregulation in primary MG, BV2 cells, and primary macrophages in vitro. Taken altogether, our results suggest that the induction of HO-1 by 4-EG in the CNS of EAE may contribute to its protective effect in EAE.

## Conclusions

Our work demonstrated for the first time that 4-EG conferred protection against autoimmune disease EAE. We showed that 4-EG suppressed pathogenic Th1/Th17 differentiation in the periphery and repressed MG activation and Th1/Th17 infiltration in the CNS of EAE. In addition, we observed 4-EG suppressed MMP3 and MMP9 production that might subsequently lessen BBB disruption and repress inflammatory immune cell infiltration of the CNS in EAE. Finally, we demonstrated that 4-EG was capable of inducing HO-1 expression that could offer anti-oxidant and anti-inflammatory effects in EAE. In summary, our study suggests that 4-EG, a natural compound, could be developed into a therapeutic agent for the treatment of MS/EAE through its immunomodulatory effects on the amelioration of disease pathogenesis.

## Supplementary Information

**Additional file 1: Table S1.** Distribution of tissues from C57BL/6 EAE mice for various experiments.

**Additional file 2: Figure S1.** Gating strategy of flow cytometry analysis. (A and B) Mononuclear cells isolated from the (A) brain and (B) spinal cord of vehicle- and 4-EG-treated C57BL/6 EAE mice were subjected to surface staining of CD4 in the presence of 7-AAD followed by intracellular staining of IFNγ, IL-17, or GM-CSF. CD4^+^ cells were gated, and 7-AAD negative live cells were then gated followed by singlet gating. Isotype controls (Iso) were used to determine CD4^+^ T cells positive for the intracellular expression of IFNγ, IL-17, or GM-CSF. (C) Splenocytes differentiated into Th1 or Th17 conditions were subjected to surface staining of CD4 in the presence of 7-AAD followed by intracellular staining of IFNγ, IL-17, or GM-CSF. CD4^+^ T cells were gated, and 7-AAD negative live cells were then gated followed by singlet gating. Iso were used as a negative control to determine CD4^+^ T cells positive for the intracellular expression of IFNγ, IL-17, or GM-CSF.

**Additional file 3: Figure S2.** The dose effects of 4-EG in chronic EAE. (A) C57BL/6 mice were immunized with MOG_35-55_ and i.p. injected with vehicle or different doses of 4-EG (50, 100, and 200 mg/kg, n=6/group) daily starting from day 3 post-immunization. The clinical score of EAE animals was followed for 30 days. Statistical significance of EAE clinical score was determined as *p<0.05 and ***p<0.001 by two-way ANOVA test. (B) EAE incidence, mortality, onset of disease, maximum score, and cumulative score were also assessed. Statistical significance was determined as *p<0.05 and ***p<0.001 by Mann-Whitney *U* test.

**Additional file 4: Figure S3.** The dose effect of 4-EG on Th1 and Th17 cell viability. Naïve splenocytes were polarized into Th1 and Th17 conditions in the presence or absence of different concentrations of 4-EG (200 and 300 μM). After 48 or 72 hours, (A) Th1 and (B) Th17 cells (n=3/group/time point) were collected and subjected to 7-AAD staining to evaluate cell viability by flow cytometery analysis. Statistical significance was determined as *p<0.05 and ***p<0.001 by one-way ANOVA test.

## Data Availability

The datasets of the current study are available from the corresponding author on a reasonable request.

## Abbreviations

MS: Multiple sclerosis
CNS: Central nervous system
EAE: Experimental autoimmune encephalomyelitis
MG: Microglia
4-EG: 4-Ethylguaiacol
BBB: Blood-brain barrier
CFA: Complete Freund’s adjuvant
PBS: Phosphate-buffered saline
7-AAD: 7-Aminoactinomycin D
NQO1: NAD(P)H quinone dehydrogenase 1
Gclc: Glutamate-cysteine ligase catalytic subunit
HO-1: Heme oxygenase-1
TJP: Tight junction protein
