# Supplementary figures and images for: 4-Ethylguaiacol modulates neuroinflammation and Th1/Th17 differentiation to ameliorate disease severity in experimental autoimmune encephalomyelitis
Source: J Neuroinflammation. 2021 May 11;18:110. doi: 10.1186/s12974-021-02143-w (PMC8111955; doi:10.1186/s12974-021-02143-w)

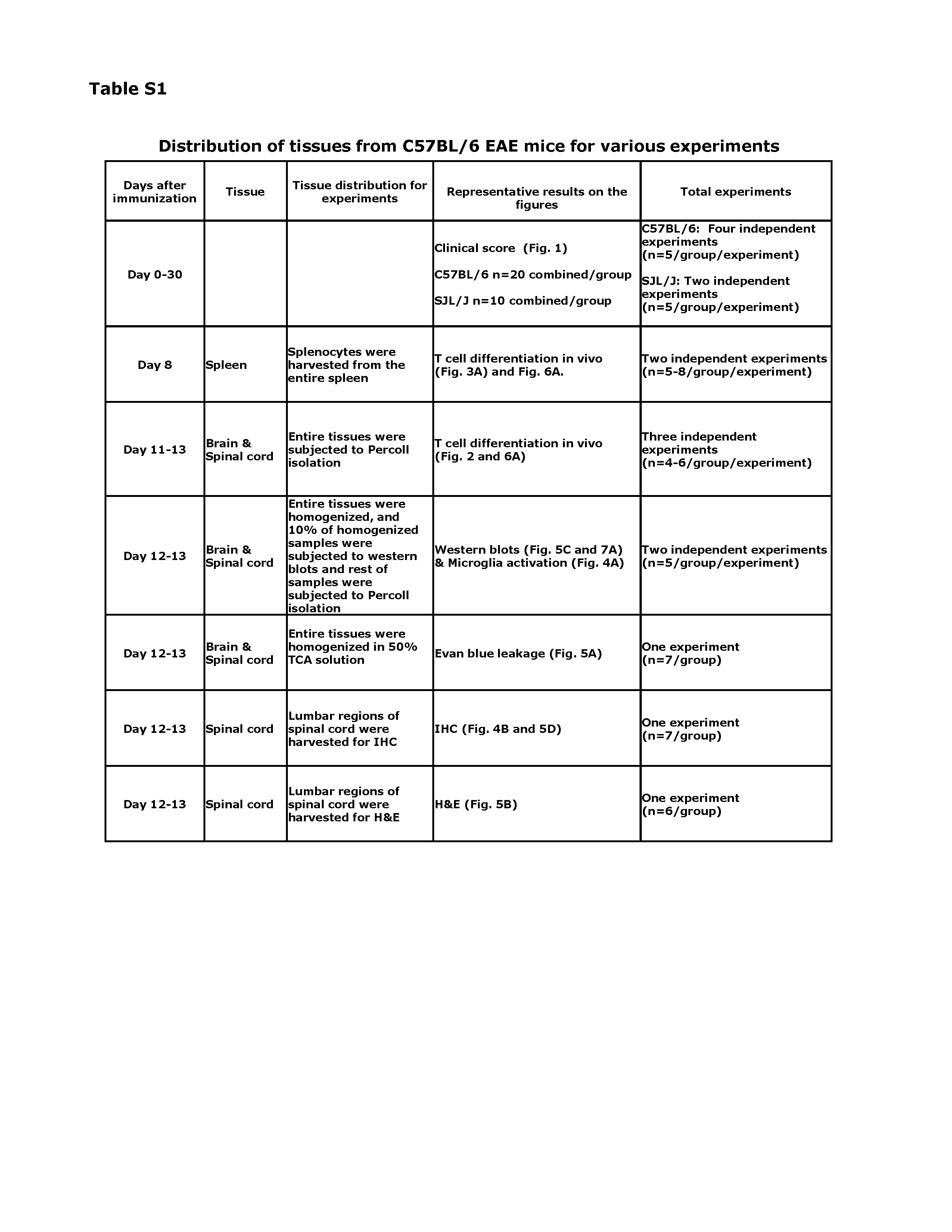

Supplement: Supplementary file 1 — Additional file 1: Table S1. Distribution of tissues from C57BL/6 EAE mice for various experiments. [file 12974_2021_2143_MOESM1_ESM.tif]

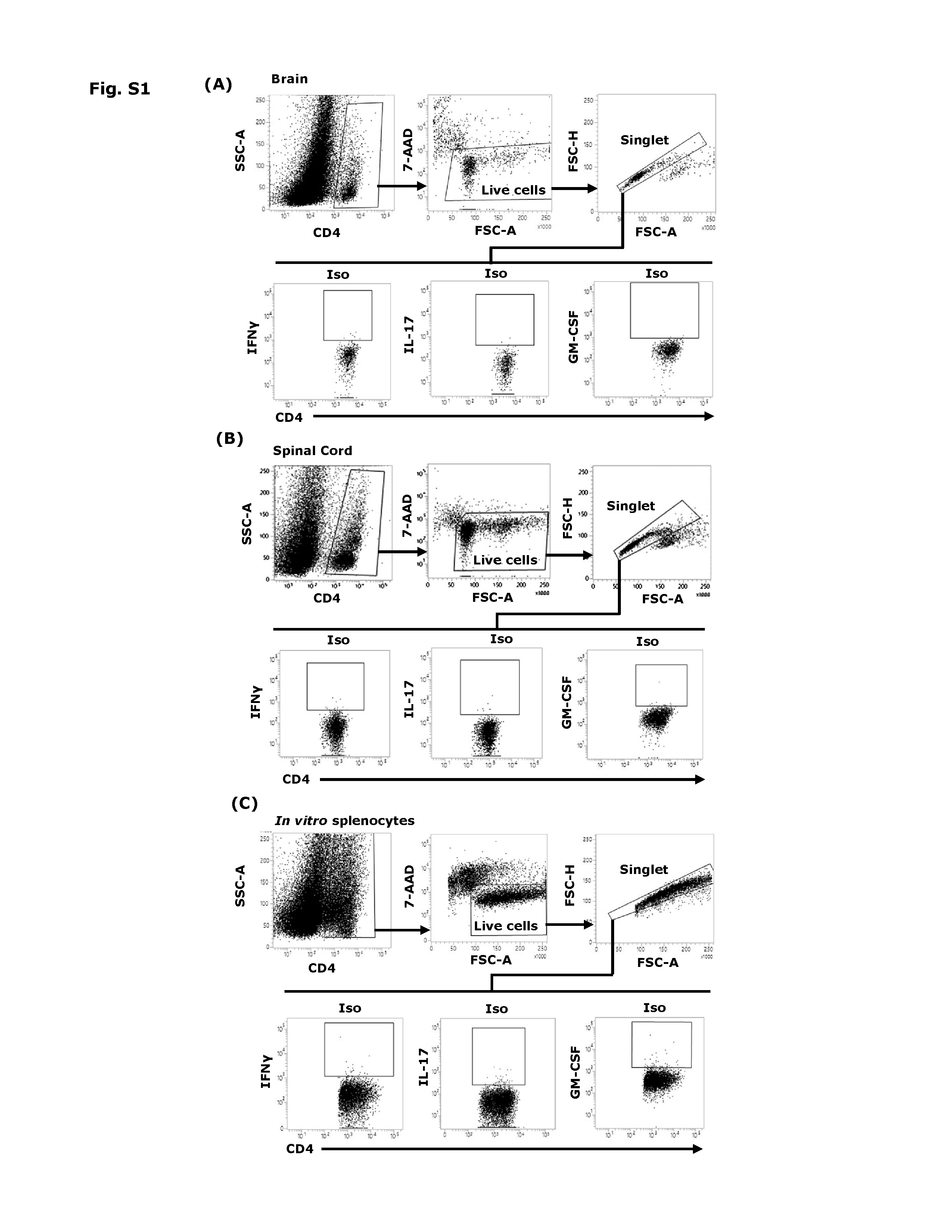

Supplement: Supplementary file 2 — Additional file 2: Figure S1. Gating strategy of flow cytometry analysis. (A and B) Mononuclear cells isolated from the (A) brain and (B) spinal cord of vehicle- and 4-EG-treated C57BL/6 EAE mice were subjected to surface staining of CD4 in the presence of 7-AAD followed by intracellular staining of IFNγ, IL-17, or GM-CSF. CD4+ cells were gated, and 7-AAD negative live cells were then gated followed by singlet gating. Isotype controls (Iso) were used to determine CD4+ T cells positive for the intracellular expression of IFNγ, IL-17, or GM-CSF. (C) Splenocytes differentiated into Th1 or Th17 conditions were subjected to surface staining of CD4 in the presence of 7-AAD followed by intracellular staining of IFNγ, IL-17, or GM-CSF. CD4+ T cells were gated, and 7-AAD negative live cells were then gated followed by singlet gating. Iso were used as a negative control to determine CD4+ T cells positive for the intracellular expression of IFNγ, IL-17, or GM-CSF. [file 12974_2021_2143_MOESM2_ESM.tif]

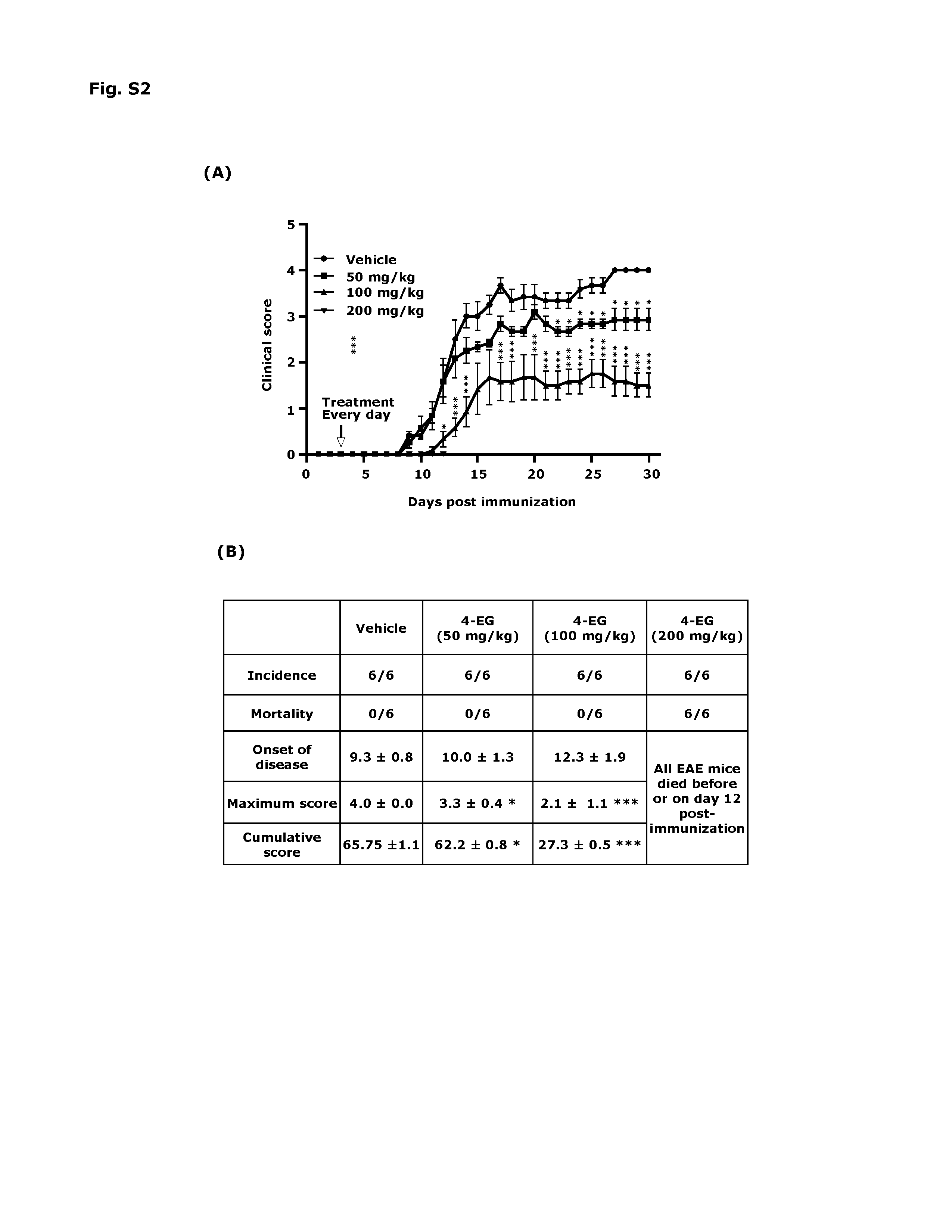

Supplement: Supplementary file 3 — Additional file 3: Figure S2. The dose effects of 4-EG in chronic EAE. (A) C57BL/6 mice were immunized with MOG35-55 and i.p. injected with vehicle or different doses of 4-EG (50, 100, and 200 mg/kg, n=6/group) daily starting from day 3 post-immunization. The clinical score of EAE animals was followed for 30 days. Statistical significance of EAE clinical score was determined as *p<0.05 and ***p<0.001 by two-way ANOVA test. (B) EAE incidence, mortality, onset of disease, maximum score, and cumulative score were also assessed. Statistical significance was determined as *p<0.05 and ***p<0.001 by Mann-Whitney U test. [file 12974_2021_2143_MOESM3_ESM.tif]

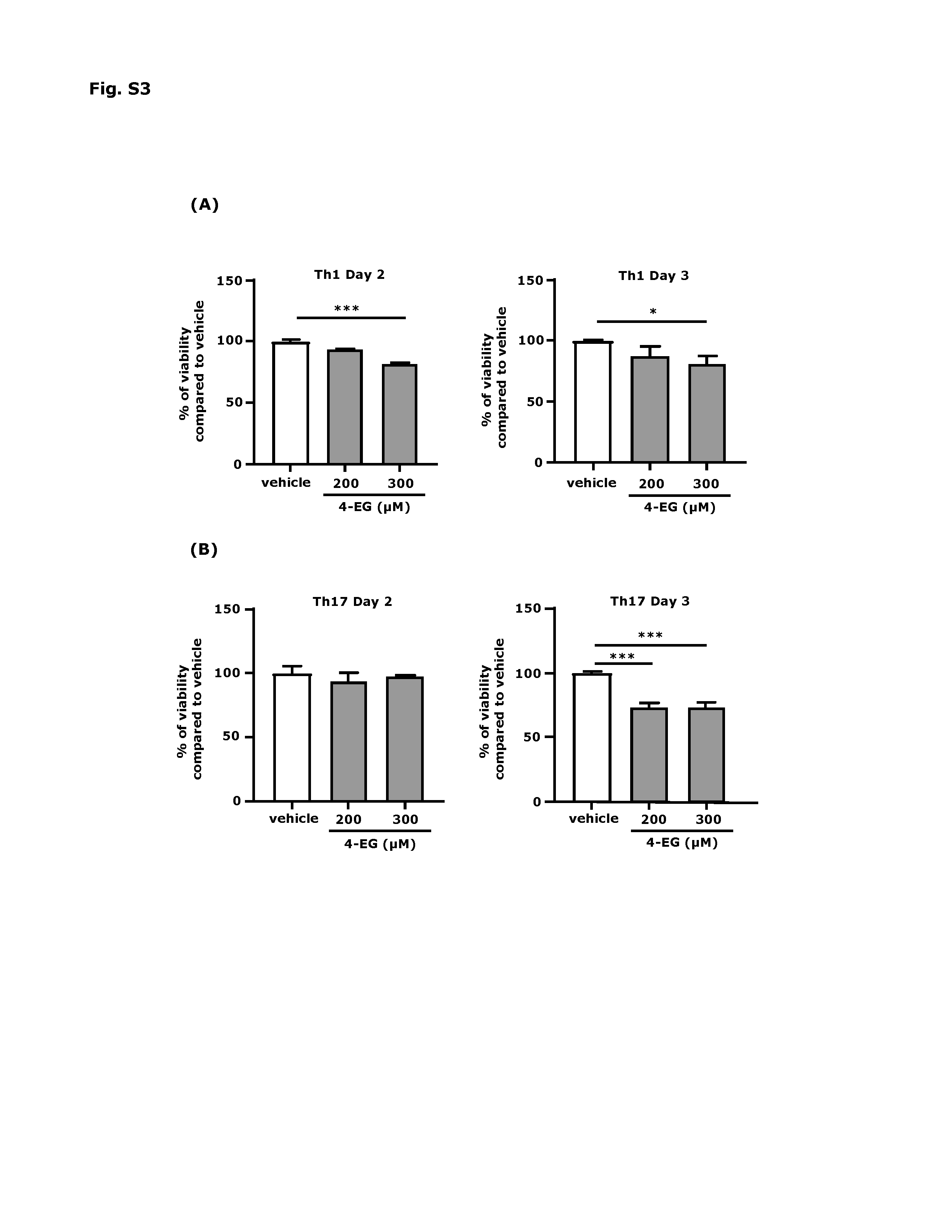

Supplement: Supplementary file 4 — Additional file 4: Figure S3. The dose effect of 4-EG on Th1 and Th17 cell viability. Naïve splenocytes were polarized into Th1 and Th17 conditions in the presence or absence of different concentrations of 4-EG (200 and 300 μM). After 48 or 72 hours, (A) Th1 and (B) Th17 cells (n=3/group/time point) were collected and subjected to 7-AAD staining to evaluate cell viability by flow cytometery analysis. Statistical significance was determined as *p<0.05 and ***p<0.001 by one-way ANOVA test. [file 12974_2021_2143_MOESM4_ESM.tif]
